# Inflammatory factors and risk of meningiomas: a bidirectional mendelian-randomization study

**DOI:** 10.3389/fnins.2023.1186312

**Published:** 2023-06-22

**Authors:** Zhiyun Zhang, Shengnan Wang, Fei Ren, Laiyu Yang, Haoqun Xie, Lin Pan, Yifan Li, Bingcheng Yu, Yifan Yang, Haoyi Su, Youqi Chen, Chuyi Zhang, Hongyu Chen, Wenzhuo Yang, Nan An, Yang Bai

**Affiliations:** ^1^Department of Neurosurgery, The First Hospital of Jilin University, Changchun, China; ^2^Department of Plastic and Reconstructive Surgery, The First Hospital of Jilin University, Changchun, China; ^3^Department of Neurology, The First Hospital of Jilin University, Changchun, China; ^4^The Second Hospital of Jilin University, Changchun, Jilin, China; ^5^Zhongshan School of Medicine, Sun Yat-sen University, Guangzhou, China; ^6^Department of Neurosurgery, Sun Yat-sen University Cancer Center, Guangzhou, China

**Keywords:** meningioma, inflammation, risk, Mendelian randomization (MR), cytokines

## Abstract

**Background:**

Meningiomas are one of the most common intracranial tumors, and the current understanding of meningioma pathology is still incomplete. Inflammatory factors play an important role in the pathophysiology of meningioma, but the causal relationship between inflammatory factors and meningioma is still unclear.

**Method:**

Mendelian randomization (MR) is an effective statistical method for reducing bias based on whole genome sequencing data. It’s a simple but powerful framework, that uses genetics to study aspects of human biology. Modern methods of MR make the process more robust by exploiting the many genetic variants that may exist for a given hypothesis. In this paper, MR is applied to understand the causal relationship between exposure and disease outcome.

**Results:**

This research presents a comprehensive MR study to study the association of genetic inflammatory cytokines with meningioma. Based on the results of our MR analysis, which examines 41 cytokines in the largest GWAS datasets available, we were able to draw the relatively more reliable conclusion that elevated levels of circulating TNF-β, CXCL1, and lower levels of IL-9 were suggestive associated with a higher risk of meningioma. Moreover, Meningiomas could cause lower levels of interleukin-16 and higher levels of CXCL10 in the blood.

**Conclusion:**

These findings suggest that TNF-β, CXCL1, and IL-9 play an important role in the development of meningiomas. Meningiomas also affect the expression of cytokines such as IL-16 and CXCL10. Further studies are needed to determine whether these biomarkers can be used to prevent or treat meningiomas.

## Introduction

Meningiomas are brain tumors that occur in the meninges surrounding the brain and spinal cord (Brastianos et al., 2019). They are one of the most common primary intracranial tumors of the central nervous system (CNS) and are second only to glioma in incidence. The vast majority of meningiomas are benign and are classified as World Health Organization (WHO) grade I; those of WHO grade II are more aggressive; and a very small percentage are malignant (only 1 to 3%) and they belong to WHO grade III (Buerki et al., 2018). Benign meningiomas can be completely cured by surgery and radiotherapy, while malignant meningiomas have a higher frequency of local invasion, recurrence, and metastasis, and the treatment options are extremely limited (Maggio et al., 2021).

The majority of meningiomas are located outside the blood–brain barrier (BBB), rendering them more vulnerable to systemic immunology and inflammation compared to structures within the BBB (von Spreckelsen et al., 2022). A retrospective study revealed elevated levels of TNF-α in meningioma patients, which induces inflammatory damage, triggers inflammatory responses, and promotes the release of pro-inflammatory factors like IL-6 (Zheng et al., 2022). Earlier investigations have demonstrated that increased IL-10 within the tumor microenvironment, including meningiomas, is associated with a poorer prognosis (Singh et al., 2019; Manjunath et al., 2022), highlighting the crucial role of inflammatory factor regulation in the pathophysiology of meningioma. Cytokines have been identified as reliable screening targets for inflammation and pain in meningiomas, with potential diagnostic and therapeutic applications (Shamsdin et al., 2019). However, these studies have focused on a limited number of inflammatory factors and have not accounted for the influence of other physical factors on altered inflammatory factor levels. Hence, it is essential to ascertain whether changes in inflammatory factors contribute to tumorigenesis or if the tumor itself modifies the microenvironment, leading to variations in inflammatory factors. Given the incomplete understanding of the etiology of malignant meningiomas, investigating the precise nature of the interaction between inflammatory factors and meningiomas holds significant clinical importance.

To establish a causal relationship between exposure to inflammatory cytokines and the development of meningiomas, we can employ Mendelian randomization (MR). MR is an observational study design that leverages genetic variants as instrumental variables to estimate the causal effect of risk factors on health outcomes. Unlike traditional multivariable observational analyses, MR is less susceptible to confounding variables and measurement errors, and avoids bias arising from reverse causality. As a result, MR has become a reliable method to obtain robust estimates for the causal impact of various risk factors on health outcomes, often yielding results similar to those obtained from randomized controlled trials (RCTs) when available (Davey Smith and Hemani, 2014).

In our study, we conducted a bidirectional Mendelian randomization analysis using genetic variations as instrumental variables to assess the causal relationship between alterations in inflammatory cytokine levels and the risk of developing meningiomas (Bouras et al., 2022). We found no evidence of a link between genetically predicted inflammatory variables and levels of potential confounders. Thus, by assuming that the connection between genetic variants and meningiomas exclusively operates through exposure, Mendelian randomization analysis can be employed to ascertain the causal influence of inflammatory factors on the risk of developing meningiomas.

## Methods

**Figure 1:**
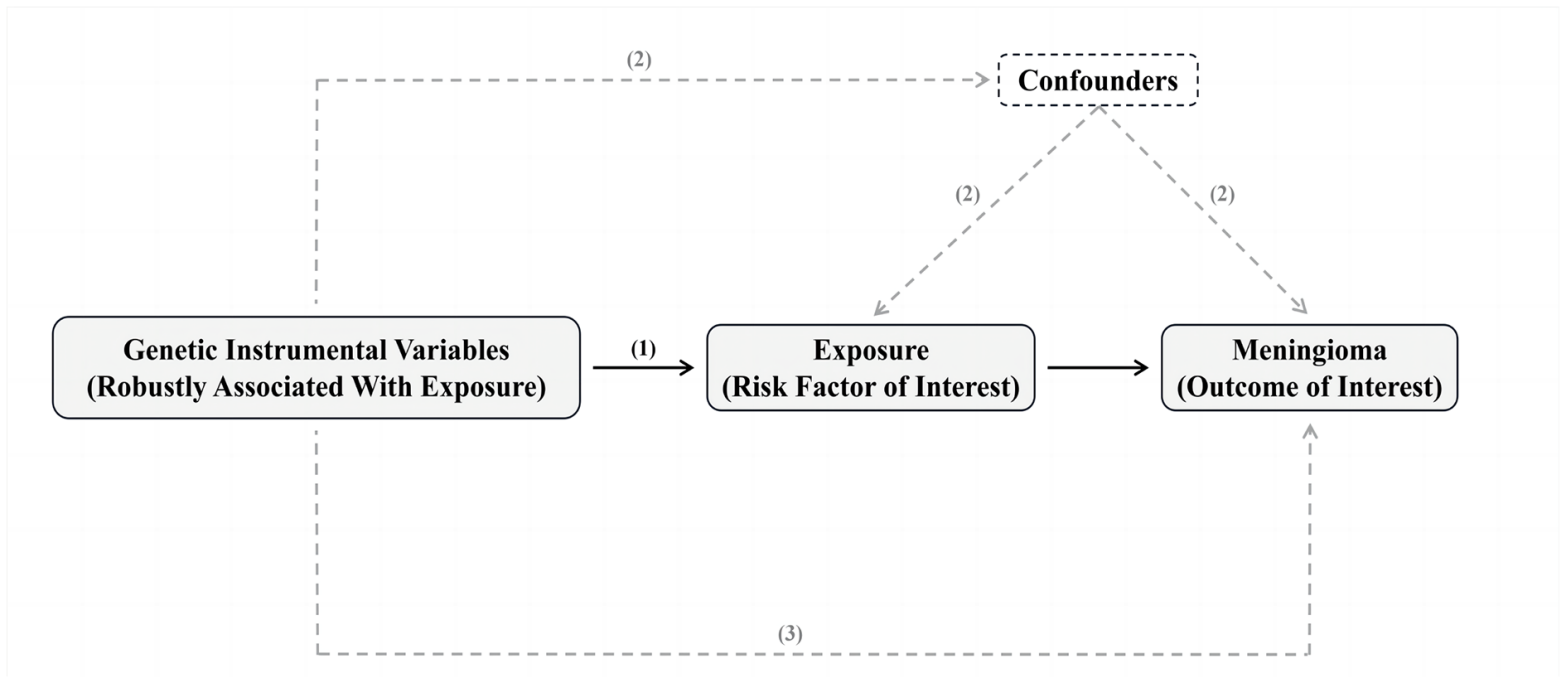
Summary of Mendelian randomization and its assumptions. The assumptions underlying Mendelian randomization (MR) analysis are as follows: (1) the genetic instrumental variable(s) should be strongly associated with the exposure (risk factor of interest); (2) there should be no confounding variables that influence both the risk factor and the outcome, and these variables should not be associated with the genetic instrument associated with risk factor and outcome either; and (3) there should be no other pathways from the genetic instrument to the outcome other than through the risk factor of interest. In practice, the last assumption is often violated due to horizontal pleiotropy, where the genetic instruments affect other factors that independently influence the outcome. This can result in biased MR estimates, either overestimating or underestimating the true effect of the risk factor on the outcome. There are various statistical methods available for estimating causal MR effects. The most intuitive method involves taking the ratio of “the association of genetic instruments with the outcome” and “the association of genetic instruments with the risk factor.” Valid MR estimates can be obtained using two independent samples, where one sample is used to assess the association of the genetic instrument with the outcome and the other sample is used to assess the association of the genetic instrument with the risk factor. The advantage of this two-sample approach is the potential to use publicly available genome-wide data to obtain large sample sizes and apply novel methods to test for horizontal pleiotropy. Please refer to the Methods section and the ESM for a detailed explanation of these methods.

MR is based on three hypotheses: (1) the genetic instrumental variable (s) should be strongly associated with the exposure (risk factor of interest); (2) there should be no confounding variables that influence both the risk factor and the outcome, and these variables should not be associated with the genetic instrument associated with risk factor and outcome either; and (3) there should be no other pathways from the genetic instrument to the outcome other than through the risk factor of interest (Bowden and Holmes, 2019; Figure 1).

### Summary statistics source

In this bidirectional Mendelian randomization study, the first step was to select appropriate genetic variants from publicly available genome-wide association study (GWAS) databases. SNPs were selected as IVs from GWAS databases for exposure and outcome. The SNPs associated with inflammatory factors were obtained from a study of 8,293 individuals that included 41 cytokines and growth factors (Ahola-Olli et al., 2017). Summary statistics for meningiomas were obtained from the UK Biobank, which included 307 cases and 456,041 controls of European ancestry, and a generalized linear mixed model (GLMM)-based method named (fastGWA-GLMM) was utilized with adjustments for covariates (Rusk, 2018). To prevent population stratification bias from confounding the findings, all SNPs and their accompanying pooled data were restricted to populations of European ancestry in this study (Jiang et al., 2021). Table 1 summarizes details about cytokines based on summary-level data from genome-wide association studies (GWAS).

**Table 1:** The sample size for each cytokine analyzed in this study acquired from the GWAS.

Cytokines	Abbreviation	Sample size	Number
Cutaneous T-cell attracting (CCL27)	CTACK	3,631	GCST004420
Beta nerve growth factor	βNGF	3,531	GCST004421
Vascular endothelial growth factor	VEGF	7,118	GCST004422
Macrophage migration inhibitory factor (glycosylation-inhibiting factor)	MIF	3,494	GCST004423
TNF-related apoptosis inducing ligand	TRAIL	8,186	GCST004424
Tumor necrosis factor-beta	TNFβ	1,559	GCST004425
Tumor necrosis factor-alpha	TNFα	3,454	GCST004426
Stromal cell-derived factor-1 alpha (CXCL12)	SDF1α	5,998	GCST004427
Stem cell growth factor beta	SCGFβ	3,682	GCST004428
Stem cell factor	SCF	8,290	GCST004429
Interleukin-16	IL-16	3,483	GCST004430
Regulated on activation, normal T cell expressed and secreted (CCL5)	RANTES	3,421	GCST004431
Platelet derived growth factor BB	PDGFbb	8,293	GCST004432
Macrophage inflammatory protein-1β (CCL4)	MIP1β	8,243	GCST004433
Macrophage inflammatory protein-1α (CCL3)	MIP1α	3,522	GCST004434
Monokine induced by interferon-gamma (CXCL9)	MIG	3,685	GCST004435
Macrophage colony-stimulating factor	MCSF	840	GCST004436
Monocyte specific chemokine 3 (CCL7)	MCP3	843	GCST004437
Monocyte chemotactic protein-1 (CCL2)	MCP1	8,293	GCST004438
Interleukin-12p70	IL-12p70	8,270	GCST004439
Interferon gamma-induced protein 10 (CXCL10)	IP10	3,685	GCST004440
Interleukin-18	IL-18	3,636	GCST004441
Interleukin-17	IL-17	7,760	GCST004442
Interleukin-13	IL-13	3,557	GCST004443
Interleukin-10	IL-10	7,681	GCST004444
Interleukin-8 (CXCL8)	IL-8	3,526	GCST004445
Interleukin-6	IL-6	8,189	GCST004446
Interleukin-1 receptor antagonist	IL1ra	3,638	GCST004447
Interleukin-1-beta	IL-1β	3,309	GCST004448
Hepatocyte growth factor	HGF	8,292	GCST004449
Interleukin-9	IL-9	3,634	GCST004450
Interleukin-7	IL-7	3,409	GCST004451
Interleukin-5	IL-5	3,364	GCST004452
Interleukin-4	IL-4	8,124	GCST004453
Interleukin-2 receptor, alpha subunit	IL2rα	3,677	GCST004454
Interleukin-2	IL-2	3,475	GCST004455
Interferon-gamma	IFN-γ	7,701	GCST004456
Growth regulated oncogene-α (CXCL1)	GROα	3,505	GCST004457
Granulocyte colony-stimulating factor	GCSF	7,904	GCST004458
Basic fibroblast growth factor	bFGF	7,565	GCST004459
Eotaxin (CCL11)	Eotaxin	8,153	GCST004460

### Instrumental variables selection

We selected SNPs strongly associated with inflammatory factors, with genome-wide significance (*P*-value < 5 × 10^–8^), as potential IVs (Burgess and Thompson, 2011). After that, we need to remove linkage disequilibrium (LD). Setting the threshold as r2 < 0.001, kb = 5,000, and removing the SNPs with r2 greater than 0.001 with the most significant SNP within 5,000 kb. Only 10 inflammatory cytokines had more than two independent SNPs at the *P*-value < 5 × 10^–8^ level after reconciling the selected SNPs with the resulting data. Therefore, we widen the threshold to *P*-value < 5 × 10^–6^ to select eligible instrumental variables. Through the above steps, we obtained 41 kinds of inflammatory factors. Due to the lowered significance threshold, IVs with F-statistics less than 10 were considered weak instrumental variables and would be excluded from our study. To comply with the law of Mendelian randomization, we will also screen the target SNPs to exclude SNPs associated with the results. Finally, the effect alleles of the genetic variants were coordinated in the exposure and outcome of GWAS, Supplementary Tables S1–S42.

### Data analysis

In this study, we primarily used the inverse variance weighting (IVW) method to estimate the causal effect of exposure on the outcome, which required SNPs to fully comply with the three principles of MR studies to obtain correct causal estimates (Wang et al., 2022). And the method will provide the most accurate results when the selected SNPs are all valid IVs. We also applied several complementary methods, including the weighted median (WM) method, and MR Egger regression, to estimate the causal associations under different conditions (Bowden et al., 2016). The WM method uses the median MR estimate as the causal estimate and has some advantages over MR Egger regression because it provides lower type I error and higher causal estimation power. MR Egger uses the reciprocal of the resulting variances as the weights for the analysis. Different from IVW, MR Egger considers the presence of an intercept term in the regression analysis. The intercept of the MR Egger regression model reveals the presence or absence of horizontal pleiotropy (*P*-value < 0.05 is considered significant) (Burgess and Thompson, 2011). When horizontal pleiotropy is present, it indicates that IVs affect outcomes independently of exposure factors, which is inconsistent with the definition of IVs. Sensitivity analyses were also performed to ensure the stability of the findings, Table 2. The Cochran Q test was used to assess heterogeneity between SNPs, Table 2. When heterogeneity was present (*P*-value < 0.05), certain SNPs with small *p*-values needed to be excluded or a random-effects model was used directly to assess the MR effect. Finally, we performed the “leave-one-out” analysis to test the stability of the results, Supplementary Figures S1–S5. The packages ‘TwoSampleMR’ in R version 4.2.2 were used for the analysis.

**Table 2:** Heterogeneity test of the IVW and MR egger analyses and pleiotropy test (egger intercept).

Exposure	Outcome	Methods	Cochran’s Q	*Q*-value	*P*-value (Pleiotropy test)
TNF-β (Tumor necrosis factor-β)	Meningiomas	MR egger	1.952	0.377	0.452
		Inverse variance weighted	2.809	0.422	
Interleukin-9	Meningiomas	MR egger	1.033	0.905	0.949
		Inverse variance weighted	1.038	0.959	
CXCL1 (Growth regulated oncogene-α)	Meningiomas	MR egger	7.452	0.383	0.617
		Inverse variance weighted	8.686	0.369	
Meningiomas	Interleukin-16	MR egger	2.796	0.947	0.102
		Inverse variance weighted	6.208	0.719	
Meningiomas	Interferon gamma-induced protein 10 (CXCL10)	MR egger	7.491	0.485	0.628
		Inverse variance weighted	7.745	0.560	

## Results

Genetically predicted systemic inflammatory regulators are associated with meningiomas, as evidenced by the following results. The higher tumor necrosis factor-beta (TNF-β) (OR = 1.351, 95% CI = 1.015–1.797) and CXCL1 (Growth regulated oncogene-α) (OR = 1.291, 95% CI = 1.002–1.663) levels are associated with an increased risk of meningiomas using IVW methods, Table 3. MR-Egger Intercept did not detect potential horizontal pleiotropy (*P*-value > 0.05). Furthermore, MR-Egger and IVW heterogeneity tests showed that there was no obvious heterogeneity (*P*-value > 0.05). Leave-one-out studies were used for sensitivity analysis and demonstrated no influence, Supplementary Figures S1, S3. Moreover, we found that higher interleukin-9 (IL-9) levels can reduce meningioma risk (OR = 0.544, 95% CI = 0.322–0.918) using IVW methods. There was no heterogeneity or horizontal pleiotropy in the results (*P*-value > 0.05). The above results are listed in Tables 2, 3.

**Table 3:** Bidirectional Mendelian randomization estimates of cytokines and meningiomas (IVW, MR-egger, weighted median, MR-PRESSO).

Exposure	Outcome	Methods	Number of SNPs	OR (95% CI)	*P*-value
TNF-β (Tumor necrosis factor-β)	Meningiomas	Inverse variance weighted	4	1.351 (1.015–1.797)	0.039
		MR egger	4	1.181 (0.789–1.767)	0.503
		Weighted median	4	1.267 (0.931–1.724)	0.132
Interleukin-9	Meningiomas	Inverse variance weighted	6	0.544 (0.322–0.918)	0.023
		MR egger	6	0.567 (0.156–2.064)	0.438
		Weighted median	6	0.576 (0.294–1.131)	0.109
CXCL1 (Growth regulated oncogene-α)	Meningiomas	Inverse variance weighted	10	1.291 (1.002–1.663)	0.048
		MR egger	10	1.127 (0.636–1.997)	0.693
		Weighted median	10	1.291 (0.950–1.753)	0.102
				BETA (95% CI)	
Meningiomas	Interleukin-16	Inverse variance weighted	10	−0.037 (−0.069 ~ −0.004)	0.027
		MR egger	10	−0.078 (−0.133 ~ −0.023)	0.023
		Weighted median	10	−0.039 (−0.084 ~ 0.005)	0.082
Meningiomas	Interferon gamma-induced protein 10 (CXCL10)	Inverse variance weighted	10	0.036 (0.003–0.068)	0.033
		MR egger	10	0.024 (−0.030–0.079)	0.402
		Weighted median	10	0.031 (−0.014–0.076)	0.182

Similarly, we found an association between genetically predicted meningiomas and cytokine levels. Genetically predicted meningiomas were associated with levels of interleukin-16 (IL-16) (BETA = −0.037, 95% CI = −0.069 ~ −0.004) and interferon gamma-induced protein 10 (IP10) (BETA = 0.036, 95% CI = 0.003–0.068) using IVW methods. There was no evidence of pleiotropy and heterogeneity observed in these results. The above results are summarized in Tables 2, 3. The Figures of Leave-one-out Analysis, Scatter Plot, Funnel Plot, and Forest Plot are listed in Supplementary material.

## Discussion

Meningiomas are one of the most common intracranial tumors. Most meningiomas occur intracranially, and a small proportion occurs in the spinal cord. Meningiomas usually grow gradually, with many tumors appearing in inaccessible places (Buerki et al., 2018; Hou et al., 2021). This sporadic behavior creates a therapeutic challenge for clinicians, as it makes it difficult to achieve complete and complete tumor removal, which in turn often leads to postoperative recurrences (Maggio et al., 2021). Inflammation in meningioma is an important part of the pathogenesis and progression. Studies have shown that in response to various stimuli and signals, circulating (systemic) immune cells can migrate out of the cerebral vasculature and into the perivascular space and brain parenchyma (Domingues et al., 2016). Most meningiomas occur outside the blood–brain barrier (BBB) and can be infiltrated by different cell types, mainly immune cells, making them more susceptible to systemic immunity and inflammation than structures inside the BBB. Some meningioma variants have also been shown to be associated with systemic inflammatory syndromes (Polyzoidis et al., 2015). Other studies have shown that the proportion of immune cells changes significantly during the development of meningiomas, suggesting that meningiomas also affect the immune response process (Sahab-Negah and Gorji, 2020). However, reverse causality and residual confounding are common biases in these traditional observational studies. Whether these changes in inflammatory modulators cause meningiomas or are a response to meningiomas remains to be investigated. Therefore, there is an urgent need for a more comprehensive understanding of the pathogenesis of meningioma, which could lead to greater advances in therapeutic approaches.

Meanwhile, by using genetic variation as an instrumental variable that can alter exposure to instrumental variables, MR studies can overcome the limitations of observational studies by examining the independent conventional biases associated with observational studies (Davey Smith and Hemani, 2014). This research presents a comprehensive MR study to study the association of genetic inflammatory cytokines with meningioma. Based on the results of our MR analysis, which examines 41 cytokines in the largest GWAS datasets available, we were able to draw the relatively more reliable conclusion that elevated levels of circulating TNF-β, CXCL1, and lower levels of IL-9 were associated with a high risk of meningioma.

TNF-β is involved in regulating tumor cell proliferation, invasion, and apoptosis, and influencing the formation of the tumor microenvironment. TNF-β activates multiple signaling pathways, including NF-κB and MAPK, promoting inflammatory responses and cell survival. Additionally, TNF-beta can induce angiogenesis, providing nutrients and oxygen to the tumor (Buhrmann et al., 2019; Zhong et al., 2022). However, there is a lack of definitive research evidence regarding the association between TNF-beta and the prognosis of meningioma patients. Some studies have shown higher levels of TNF-beta in the peripheral blood of meningioma patients compared to normal individuals (Boyle-Walsh et al., 1996), which may be associated with increased tumor invasiveness, higher risk of recurrence, and enhanced resistance to treatment. Increased expression of CXCL1 is associated with tumor development and progression in meningiomas. CXCL1 is a chemokine that attracts leukocytes and other immune cells to sites of inflammation or tumors (Acharyya et al., 2012). Research has demonstrated that CXCL1 can promote the proliferation of human meningioma cells (Barbieri et al., 2006). In our study, using a Mendelian randomization approach, we also found that elevated levels of CXCL1 are associated with an increased risk of developing meningiomas. This finding supports the role of CXCL1 in meningioma pathogenesis and suggests its potential as a risk factor for the development of this tumor. The ability of CXCL1 to enhance cell proliferation provides a mechanistic explanation for its involvement in meningioma development. These findings highlight the importance of CXCL1 as a potential therapeutic target and underscore the need for further investigations to explore its precise mechanisms of action in meningioma tumorigenesis. IL-9 activates downstream signaling pathways, such as JAK/STAT and MAPK, by binding to its receptor IL-9R. In certain tumor types, IL-9 may promote tumor growth and metastasis (Angkasekwinai and Dong, 2021). In contrast, our study found that elevated levels of IL-9 in peripheral blood are associated with a decreased risk of developing meningiomas. However, the specific mechanisms of IL-9 in meningiomas require further investigation. Overall, TNF-beta may promote tumor growth and metastasis in meningiomas, while CXCL1 may be involved in tumor invasiveness and resistance to treatment. The role of IL-9 in meningiomas requires further investigation. These research findings provide an important foundation for gaining a deeper understanding of the mechanisms underlying meningioma development and for developing corresponding therapeutic strategies.

The bilateral MR analysis in this study also showed that meningiomas may not be correlated with changes in blood cytokine levels. IL-16, as a multifunctional cytokine, plays a role in regulating immune cell functions in cancer. It enhances the activity of natural killer (NK) cells and promotes cytotoxicity of T cells, thereby inhibiting tumor cell proliferation and dissemination. Additionally, IL-16 has been associated with poor prognosis in cancers such as gastric cancer, possibly due to its ability to attract other immune cells into the tumor microenvironment, including regulatory T cells, influencing tumor growth and progression (Liu et al., 2016; Xiong et al., 2022). Our research findings indicate that meningiomas lead to a decrease in IL-16 levels, although the evidence regarding the relationship between meningiomas and IL-16 is limited. We speculate that the decrease in IL-16 levels may suggest the presence of similar immunosuppressive mechanisms in the meningioma microenvironment, which contribute to tumor growth and evasion of immune surveillance. On the other hand, IP-10 (also known as CXCL10) is a chemokine involved in recruiting and activating immune cells. Elevated levels of IP-10 have been observed in various cancers and are associated with tumor progression, angiogenesis, and immune cell infiltration (Karin and Razon, 2018; Chen et al., 2020; Wu et al., 2021; Limagne et al., 2022). The increased levels of IP-10 in the blood of meningioma patients may reflect inflammatory responses to the tumor or aggregation of immune cells. In summary, the alterations in IL-16 and IP-10 levels in the blood of meningioma patients indicate complex interactions between tumors and the immune system. These findings support the importance of cytokine dysregulation and immune modulation in the pathogenesis of meningiomas. Further research is needed to elucidate the precise mechanisms underlying these cytokine changes and their impact on the development, progression, and therapeutic strategies for meningiomas.

There are several limitations to our study. Firstly, in the GWAS data for cytokines, we used a significance cut-off of *P*-value < 5 × 10^−6^ because only 10 had at least one genome-wide significant SNP at a cut-off of *P*-value < 5 × 10^−8^. Secondly, the result of our MR-Egger and Weight Median estimates were not significant. As the statistical power of the IVW method was significantly higher than other MR methods, especially MR-Egger, and the fact that we followed the strengthened requirement in the consistent β-direction of MR methods in our study, our result can also be considered significant. The third issue is that all GWAS data are from European populations and there is a shortcoming of whether our findings will be consistent across populations, which remains to be seen. The results of these studies may be influenced by other measured and unmeasured confounders, and cytokine production may be influenced by many other factors, including the cytokine network system, rather than the disease itself.

## Data availability statement

The original contributions presented in the study are included in the article/Supplementary material, further inquiries can be directed to the corresponding author.

## Author contributions

ZZ and SW conceived the ideas, designed the experiments, and wrote the manuscript. FR, LY, HX, LP, YL, BY, YY, HS, YC, CZ, HC, WY, and NA carried out experiments and analyzed experiments results. YB revised the manuscript, figures, and tables. All authors contributed to the article and approved the submitted version.

## Funding

This work was supported by the Natural Science Foundation of Jilin Province to Xing Su (YDZJ202301ZYTS081).

## Conflict of interest

The authors declare that the research was conducted in the absence of any commercial or financial relationships that could be construed as a potential conflict of interest.

## Publisher’s note

All claims expressed in this article are solely those of the authors and do not necessarily represent those of their affiliated organizations, or those of the publisher, the editors and the reviewers. Any product that may be evaluated in this article, or claim that may be made by its manufacturer, is not guaranteed or endorsed by the publisher.
