# Supplementary material for: Inflammatory factors and risk of meningiomas: a bidirectional mendelian-randomization study
Source: Front Neurosci. 2023 Jun 22;17:1186312. doi: 10.3389/fnins.2023.1186312 (PMC10325787; doi:10.3389/fnins.2023.1186312)
Supplement: Supplementary file 1 [file Data_Sheet_1.DOCX]

**Supplementary Material**

**(1) Details of each of the three two-sample MR methods that were used**

In method 1, which we considered to be the main analysis (with other methods providing important sensitivity analyses), the SNP-specific Wald ratio estimates (e.g. the ratio of log odds of Facial Aging per effect allele to change in BMI per effect allele) were combined using the inverse-variance weighted (IVW) approach. This method may be biased if any of the instrumental variables are invalid (i.e. if they influence the outcome by other pathways that are independent from the exposure of interest, known as horizontal pleiotropy)[[1]](https://paperpile.com/c/CXJUNW/x4ty). Thus, we also used weighted median (method 2) and MR-Egger (method 3) to combine the SNP specific estimates[[2]](https://paperpile.com/c/CXJUNW/iHV9). These methods seek to obtain an MR estimate that is robust to horizontal pleiotropy. The different assumptions of each method are described as below.

**Inverse Variance Weighted (IVW) Method**

The IVW method combines the SNP-specific Wald estimates (ratio of SNP on outcome to SNP on exposure) using the following formulas:

Where ***E_k_*** is the mean change in exposure level per additional effect allele of SNP k and ***D_k_*** is the mean change in outcomes (e.g. log odds of Facial Aging or levels of other Facial Aging risk factors) per additional effect allele of SNP k with standard error ***σ_Dk_***. The results using IVW were also used to estimate the intermediating effect of facial aging risk factors on the causal association between BMI and Facial Aging.

This method provides a consistent estimate of the causal effect if all genetic variants (SNPs) used as instrumental variables satisfy the instrumental variable (IV) assumptions, specifically, 1) the genetic variants are predictive of the exposure, 2) the genetic variants are independent of any confounding factors of the exposure-outcome association, and 3) the genetic variants are independent of the outcome given the exposure and confounding factors (exclusion restriction criteria). It does not test for or take account of horizontal pleiotropy, which if present would result in violation of the exclusion restriction criteria assumption and could importantly bias the estimate of causal effect.

The IVW estimate is a statistically efficient method, but can be biased even if just one genetic variant is invalid (i.e. if just one variant has horizontal pleiotropic effects). For this reason, we used weighted median method in addition to the IVW to account for the possibility of the existence of invalid genetic IVs.

**Weighted median estimator**

The weighted median estimator is the median of a distribution having estimate **β_j_** as its **P_j_ = 100(S_j_ − W_j_ /2)^th^** percentile, where **P** is the percentile for the **j^th^** ordered ratio estimate, **W_j_** is the weight given to the **j^th^** ordered ratio estimate, proportional to the inverse of the IV variance, and **S_j_** is the sum of weights up to and including the weight of the **j^th^** ordered ratio estimates, calculated using the following equation:

Weights are standardized, so that the sum of the weights **S_j_** is one. As with the simple median, this method assumes that no more than 50% of the genetic IVs are invalid. Additionally it assumes that no single IV contributes more than 50% of the weight. It is more statistically efficient than the simple median method.

**MR-Egger regression**

The MR-Egger method was developed by Bowden et al. to specifically test for horizontal pleiotropy and correct for this in MR analyses[[1]](https://paperpile.com/c/CXJUNW/x4ty). MR Egger uses a weighted linear regression of the gene–outcome coefficients **θ_j_** on the gene–exposure coefficients **δ_j_ : θ_j_ = β_0E_+ β_E_×δ_j_**, in which all the **δ_j_** associations are orientated to be positive, and the weights in the regression are the inverse-variances of the gene–outcome associations

**(2) Calculation of the proportion of mediation effect**

The proportion of the effect that is mediated by any of the potential mediators was estimated using the following equation[[3]](https://paperpile.com/c/CXJUNW/VC96):

$$E(\%)=\frac{\sum_{K=1}^{K} \beta1\times\beta2_{\text{k}}}{\sum_{K=1}^{K} \beta3+\beta1\times\beta2_{\text{k}}}$$

Taking into account the genetically determined mediator factors, we performed multivariable Mendelian randomization (MR) analysis to estimate the direct causal effect of BMI on facial aging, adjusting for the SNP-potential mediator effect. The indirect causal effect (mediation effect) mediated by the mediator factor was obtained through ***β1 × β2***, where ***β1*** is the direct causal effect of BMI on facial aging, and ***β2*** is the causal effect of the genetically determined mediator factor on facial aging adjusted for BMI. ***β3*** is the effect of BMI on facial aging adjusted for the genetically determined mediator factor.

**References**

1. [Bowden J, Davey Smith G, Haycock PC, Burgess S. Consistent Estimation in Mendelian Randomization with Some Invalid Instruments Using a Weighted Median Estimator. Genet Epidemiol. 2016;40: 304–314.](http://paperpile.com/b/CXJUNW/x4ty)

2. [Bowden J, Davey Smith G, Burgess S. Mendelian randomization with invalid instruments: effect estimation and bias detection through Egger regression. Int J Epidemiol. 2015;44: 512–525.](http://paperpile.com/b/CXJUNW/iHV9)

3. [Varbo A, Benn M, Smith GD, Timpson NJ, Tybjaerg-Hansen A, Nordestgaard BG. Remnant cholesterol, low-density lipoprotein cholesterol, and blood pressure as mediators from obesity to ischemic heart disease. Circ Res. 2015;116: 665–673.](http://paperpile.com/b/CXJUNW/VC96)

**Abbreviation:**

Mendelian randomization, MR;

Genome Wide Association Study, GWAS;

Tumor necrosis factor-beta, TNF-β;

Growth regulated oncogene-α, CXCL1;

Interleukin-9, IL-9;

Interferon gamma-induced protein 10, CXCL10;

Interleukin-16, IL-16;

World Health Organization, WHO;

Central Nervous System, CNS;

Blood-Brain Barrier, BBB;

Tumor necrosis factor-alpha, TNF-α;

Randomized Controlled Trials, RCTs;

Single Nucleotide Polymorphism, SNP;

Inverse Variance Weighting, IVW;

Weighted Median, WM;

**Supplementary Figure 1 Leave-one-out Analysis, Scatter Plot, Funnel Plot, and Forest Plot of TNF-βon Meningiomas**

**
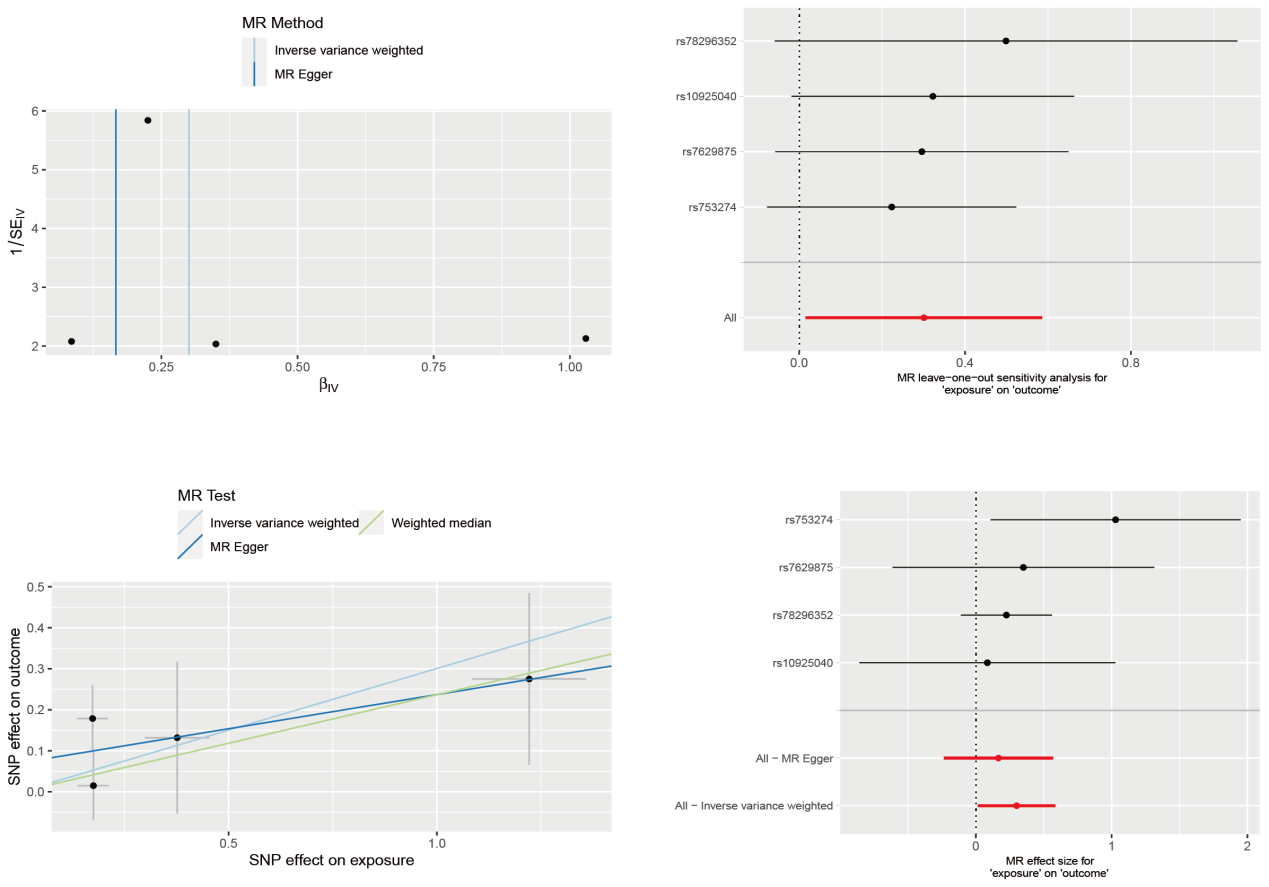
**

**Supplementary Figure 2 Leave-one-out Analysis, Scatter Plot, Funnel Plot, and Forest Plot of Interleukin-9 on Meningiomas**

**
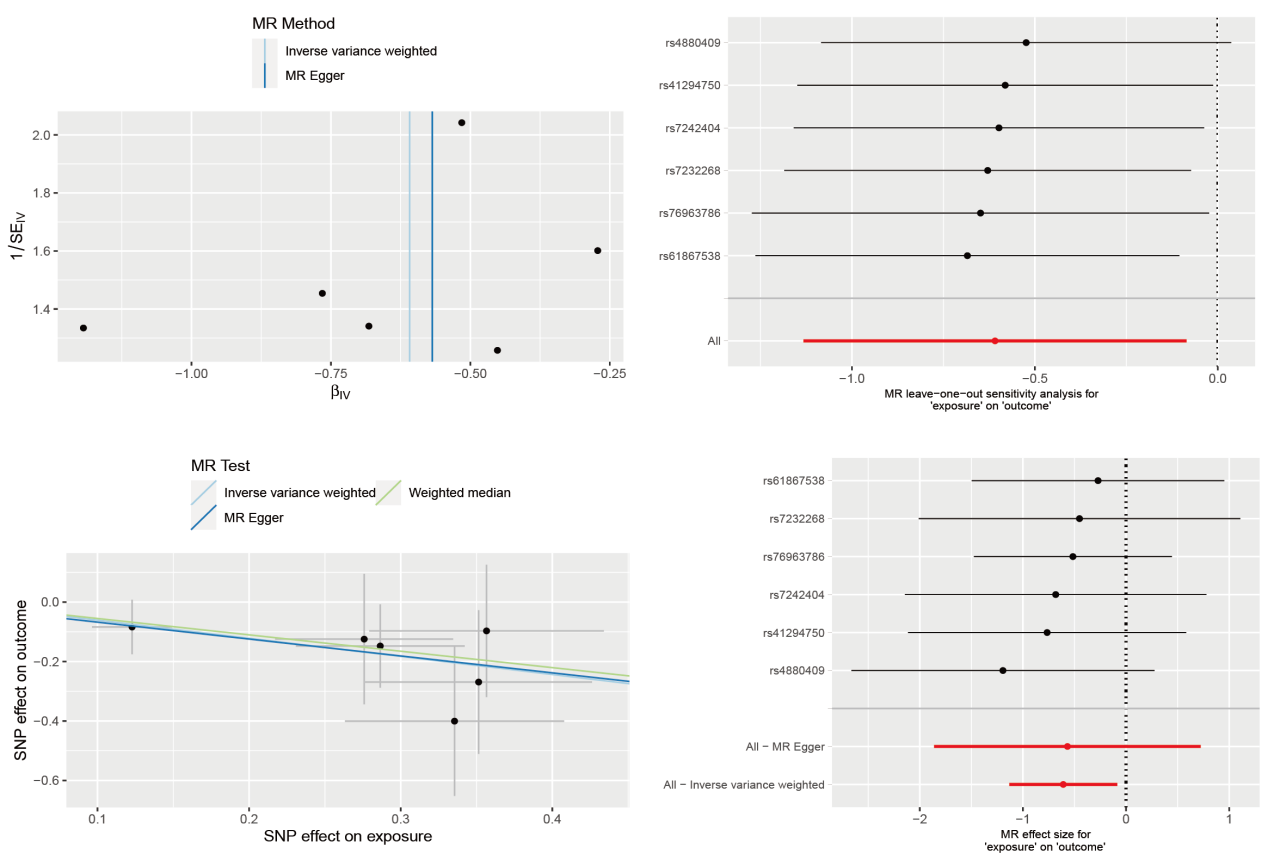
**

**Supplementary Figure 3 Leave-one-out Analysis, Scatter Plot, Funnel Plot, and Forest Plot of CXCL1 (Growth regulated oncogene-α) on Meningiomas**

**
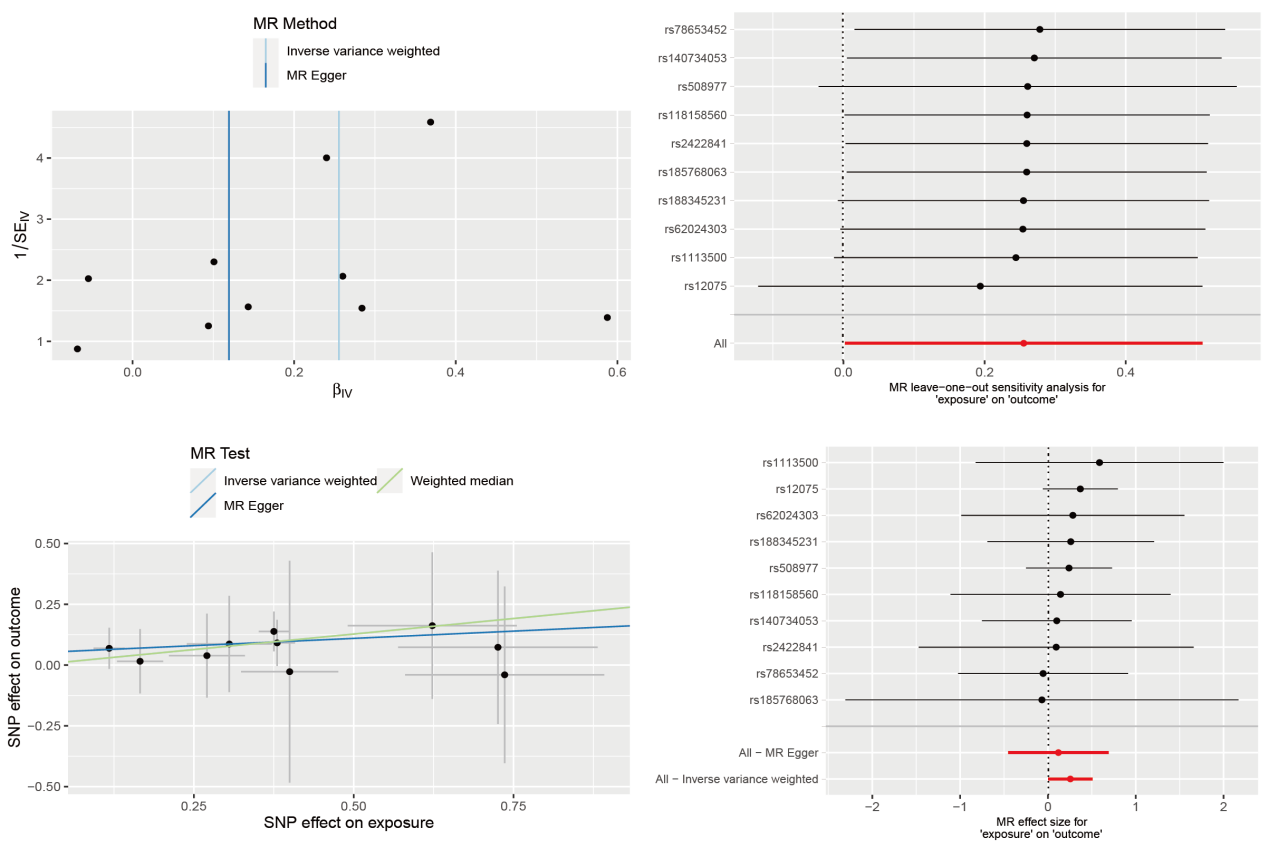
**

**Supplementary Figure 4 Leave-one-out Analysis, Scatter Plot, Funnel Plot, and Forest Plot of Meningiomas on Interleukin-16**

**
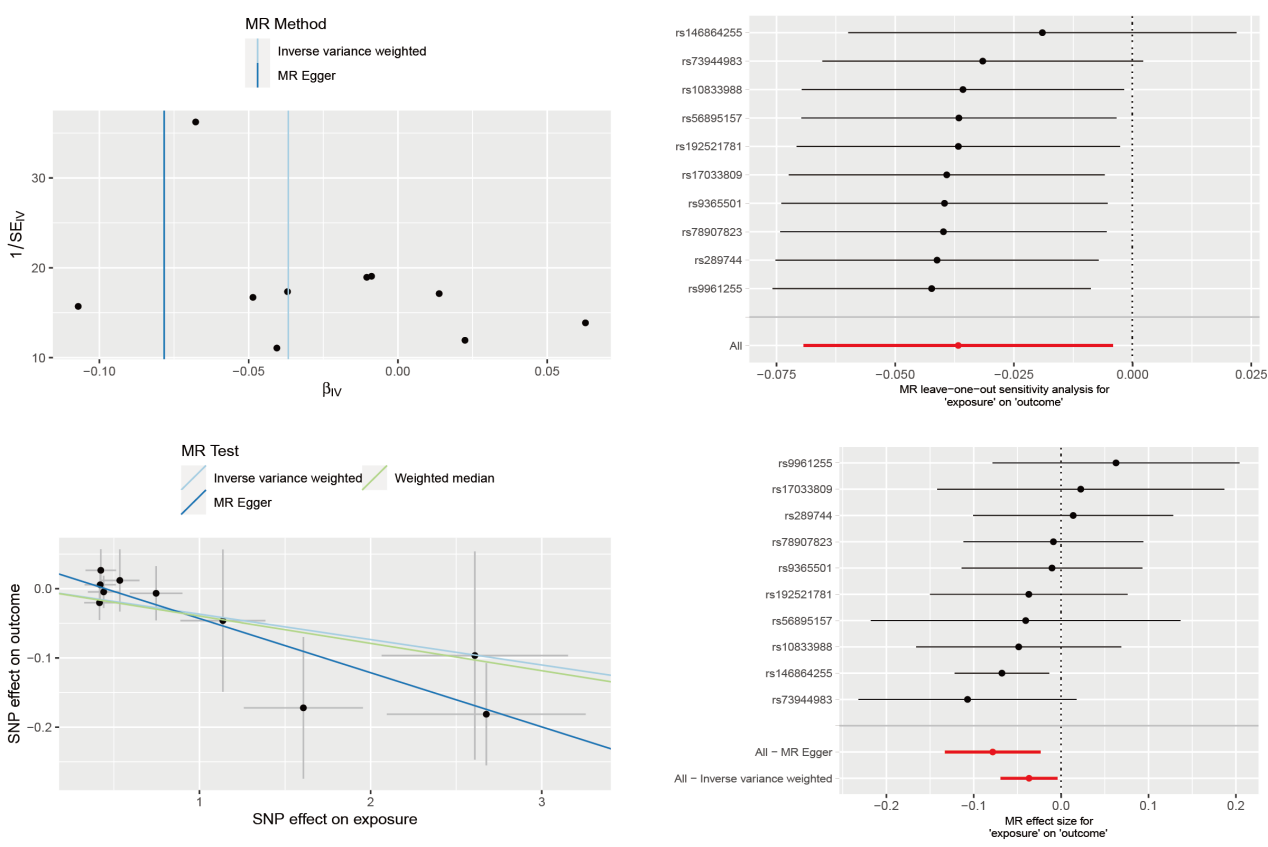
**

**Supplementary Figure 5 Leave-one-out Analysis, Scatter Plot, Funnel Plot, and Forest Plot of Meningiomas on Interferon gamma-induced protein 10 (CXCL10)**

**
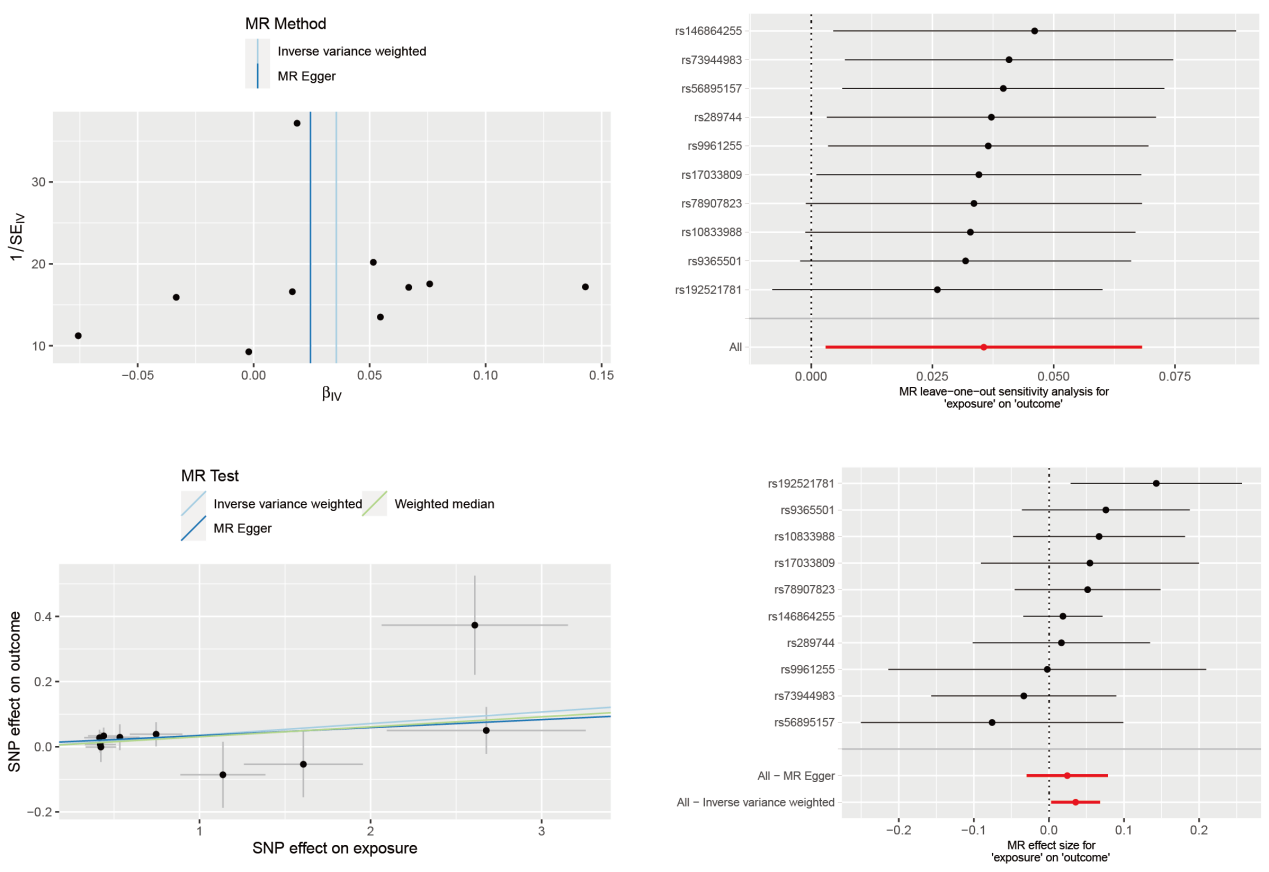
**

**Supplementary Table 1** Characteristics of the single nucleotide polymorphisms (SNP) used as instrumental variables for Cutaneous T-cell attracting (CCL27)

| **rsID** | **Effect Allele** | **Other Allele** | **BETA** | **SE** | **P Value** |
| --- | --- | --- | --- | --- | --- |
| rs113436108 | G | C | 0.5249 | 0.1119 | 3.42E-06 |
| rs116303454 | G | A | -0.383 | 0.0816 | 3.27E-06 |
| rs12438356 | C | T | -0.1548 | 0.0337 | 4.46E-06 |
| rs145902143 | G | A | 0.2838 | 0.0581 | 1.03E-06 |
| rs2070074 | G | A | -0.4467 | 0.0374 | 1.79E-32 |
| rs2731674 | G | T | 0.1333 | 0.0267 | 5.64E-07 |
| rs3766110 | C | A | 0.1287 | 0.0278 | 3.86E-06 |
| rs55764737 | C | T | -0.5313 | 0.0972 | 4.62E-08 |
| rs57338032 | G | A | -0.1583 | 0.0317 | 6.23E-07 |
| rs7333764 | C | T | -0.2773 | 0.0593 | 2.85E-06 |
| rs76395525 | G | A | -0.5277 | 0.1083 | 9.55E-07 |

**Supplementary Table 2** Characteristics of the single nucleotide polymorphisms (SNP) used as instrumental variables for Beta nerve growth factor (βNGF)

| **rsID** | **Effect Allele** | **Other Allele** | **BETA** | **SE** | **P Value** |
| --- | --- | --- | --- | --- | --- |
| rs28637706 | G | T | 0.1589 | 0.0263 | 1.42E-09 |
| rs67476890 | C | T | -0.1769 | 0.0379 | 3.13E-06 |
| rs71641308 | C | T | -0.2043 | 0.0432 | 2.30E-06 |
| rs72780728 | G | A | -0.1883 | 0.0403 | 2.99E-06 |
| rs73472576 | C | T | 0.1181 | 0.0252 | 2.69E-06 |
| rs7970581 | G | T | -0.138 | 0.0282 | 9.27E-07 |
| rs9436119 | G | A | 0.1121 | 0.0246 | 3.91E-06 |

**Supplementary Table 3** Characteristics of the single nucleotide polymorphisms (SNP) used as instrumental variables for Vascular endothelial growth factor (VEGF)

| **rsID** | **Effect Allele** | **Other Allele** | **BETA** | **SE** | **P Value** |
| --- | --- | --- | --- | --- | --- |
| rs10153304 | G | A | -0.1547 | 0.0325 | 1.94E-06 |
| rs10761731 | T | A | 0.1187 | 0.0174 | 1.01E-11 |
| rs10934631 | C | T | 0.1151 | 0.0245 | 2.47E-06 |
| rs10967186 | C | T | -0.0898 | 0.017 | 1.23E-07 |
| rs13209117 | G | A | -0.1302 | 0.0201 | 5.28E-11 |
| rs143479231 | G | A | 0.2598 | 0.0491 | 1.90E-07 |
| rs4082730 | G | A | -0.2522 | 0.0534 | 2.64E-06 |
| rs6921438 | G | A | 0.49 | 0.0175 | 2.09E-171 |
| rs7030781 | T | A | -0.1368 | 0.0173 | 2.57E-15 |
| rs73418461 | G | A | 0.2492 | 0.0521 | 1.68E-06 |
| rs8045833 | G | A | -0.108 | 0.0211 | 2.83E-07 |

**Supplementary Table 4** Characteristics of the single nucleotide polymorphisms (SNP) used as instrumental variables for Macrophage migration inhibitory factor (MIF)

| **rsID** | **Effect Allele** | **Other Allele** | **BETA** | **SE** | **P Value** |
| --- | --- | --- | --- | --- | --- |
| rs113218956 | G | A | 0.8948 | 0.1879 | 2.26E-06 |
| rs11551183 | G | C | -0.3715 | 0.0797 | 2.53E-06 |
| rs118055855 | C | T | -0.6907 | 0.15 | 4.13E-06 |
| rs12594190 | G | A | -0.1355 | 0.0267 | 3.70E-07 |
| rs13142904 | C | T | 0.223 | 0.0425 | 2.56E-07 |
| rs141009259 | C | T | 0.6178 | 0.1322 | 2.47E-06 |
| rs2330634 | G | C | -0.1556 | 0.0249 | 4.53E-10 |
| rs78098071 | C | T | 0.4867 | 0.0918 | 1.78E-07 |

**Supplementary Table 5** Characteristics of the single nucleotide polymorphisms (SNP) used as instrumental variables for TNF-related apoptosis inducing ligand (TRAIL)

| **rsID** | **Effect Allele** | **Other Allele** | **BETA** | **SE** | **P Value** |
| --- | --- | --- | --- | --- | --- |
| rs10075741 | G | C | -0.0881 | 0.0189 | 3.34E-06 |
| rs11618126 | G | A | -0.8908 | 0.1914 | 1.46E-06 |
| rs11657269 | G | A | -0.1188 | 0.026 | 4.78E-06 |
| rs11699445 | G | T | -0.0746 | 0.0161 | 3.27E-06 |
| rs13185784 | G | A | -0.0846 | 0.0183 | 3.90E-06 |
| rs13278062 | G | T | -0.0801 | 0.0157 | 3.57E-07 |
| rs138987090 | G | A | 0.7497 | 0.0752 | 4.50E-23 |
| rs146783010 | G | A | 0.6016 | 0.135 | 4.83E-06 |
| rs148051545 | C | T | 0.3921 | 0.0848 | 3.86E-06 |
| rs193112415 | C | T | 1.0421 | 0.0623 | 2.15E-62 |
| rs28431810 | G | C | 0.1195 | 0.0253 | 2.52E-06 |
| rs28521641 | T | A | 0.7019 | 0.0447 | 1.30E-56 |
| rs57396456 | C | T | 0.5626 | 0.0518 | 1.25E-27 |
| rs62093514 | C | T | -1.0618 | 0.0552 | 6.86E-82 |
| rs73039026 | C | A | 0.2999 | 0.0635 | 2.02E-06 |
| rs747324 | C | T | 0.0855 | 0.0178 | 1.61E-06 |
| rs74778900 | C | T | -0.5906 | 0.0532 | 2.59E-28 |
| rs75928541 | G | A | -0.275 | 0.0593 | 4.24E-06 |
| rs79287178 | G | A | 0.4317 | 0.0421 | 9.12E-25 |

**Supplementary Table 6** Characteristics of the single nucleotide polymorphisms (SNP) used as instrumental variables for Tumor necrosis factor-beta (TNF-β)

| **rsID** | **Effect Allele** | **Other Allele** | **BETA** | **SE** | **P Value** |
| --- | --- | --- | --- | --- | --- |
| rs10925040 | C | T | -0.1755 | 0.0373 | 2.67E-06 |
| rs75240021 | G | C | -0.3705 | 0.0772 | 1.61E-06 |
| rs753274 | C | T | 0.1736 | 0.0371 | 2.77E-06 |
| rs7629875 | G | A | -0.3766 | 0.0774 | 1.37E-06 |
| rs78296352 | G | T | -1.2215 | 0.1366 | 4.76E-21 |

**Supplementary Table 7** Characteristics of the single nucleotide polymorphisms (SNP) used as instrumental variables for Tumor necrosis factor-alpha (TNF-α)

| **rsID** | **Effect Allele** | **Other Allele** | **BETA** | **SE** | **P Value** |
| --- | --- | --- | --- | --- | --- |
| rs10834997 | G | A | 0.1247 | 0.0258 | 1.33E-06 |
| rs111332265 | G | A | 0.3766 | 0.0754 | 6.63E-07 |
| rs115669577 | G | A | -0.9889 | 0.1995 | 8.28E-07 |
| rs79105320 | G | A | -0.5605 | 0.1179 | 3.59E-06 |
| rs8121916 | C | A | -0.1306 | 0.0278 | 2.72E-06 |

**Supplementary Table 8** Characteristics of the single nucleotide polymorphisms (SNP) used as instrumental variables for Stromal cell-derived factor-1 alpha (CXCL12)

| **rsID** | **Effect Allele** | **Other Allele** | **BETA** | **SE** | **P Value** |
| --- | --- | --- | --- | --- | --- |
| rs10013755 | T | A | -0.5302 | 0.1 | 1.50E-07 |
| rs10474392 | G | A | -0.0962 | 0.0178 | 1.24E-06 |
| rs12407262 | G | A | -0.1179 | 0.0266 | 3.99E-06 |
| rs13400104 | G | A | 0.0647 | 0.0189 | 4.53E-06 |
| rs139840550 | G | A | -0.1834 | 0.0549 | 3.79E-06 |
| rs149893336 | G | A | 0.5034 | 0.1081 | 4.52E-06 |
| rs4581824 | G | T | 0.0701 | 0.0173 | 3.05E-06 |
| rs482700 | G | A | 0.0893 | 0.0203 | 1.57E-06 |
| rs67689854 | C | A | 0.0681 | 0.0195 | 3.07E-06 |

**Supplementary Table 9** Characteristics of the single nucleotide polymorphisms (SNP) used as instrumental variables for Stem cell growth factor beta (SCGF-β)

| **rsID** | **Effect Allele** | **Other Allele** | **BETA** | **SE** | **P Value** |
| --- | --- | --- | --- | --- | --- |
| rs112346514 | C | T | 0.3314 | 0.0711 | 2.37E-06 |
| rs116924815 | C | T | -0.6079 | 0.0738 | 1.74E-16 |
| rs117716477 | C | A | -0.8384 | 0.0841 | 1.34E-23 |
| rs12480722 | C | T | -0.1624 | 0.0355 | 4.72E-06 |
| rs13287050 | T | A | 0.1242 | 0.0264 | 2.20E-06 |
| rs139413256 | G | A | 0.5377 | 0.1084 | 7.04E-07 |
| rs143829871 | C | T | 0.1902 | 0.04 | 1.90E-06 |
| rs151194174 | G | A | -0.4635 | 0.0942 | 1.13E-06 |
| rs17876031 | G | A | 0.1514 | 0.0255 | 2.25E-09 |
| rs264162 | G | A | -0.1097 | 0.0234 | 2.69E-06 |
| rs34911860 | G | A | 0.3675 | 0.0789 | 3.24E-06 |
| rs4656185 | G | A | -0.205 | 0.0256 | 1.16E-15 |
| rs4737732 | G | A | 0.1147 | 0.0252 | 4.68E-06 |
| rs7762066 | C | T | -0.1389 | 0.0299 | 3.50E-06 |
| rs78217154 | C | T | -0.3997 | 0.0864 | 3.77E-06 |

**Supplementary Table 10** Characteristics of the single nucleotide polymorphisms (SNP) used as instrumental variables for Stem cell factor (SCF)

| **rsID** | **Effect Allele** | **Other Allele** | **BETA** | **SE** | **P Value** |
| --- | --- | --- | --- | --- | --- |
| rs113127926 | C | A | -0.1982 | 0.042 | 2.27E-06 |
| rs13412535 | G | A | 0.1067 | 0.0213 | 6.04E-07 |
| rs1557570 | G | T | -0.1186 | 0.017 | 2.74E-12 |
| rs1568119 | C | T | 0.5906 | 0.1129 | 1.24E-07 |
| rs1942355 | C | T | 0.0716 | 0.0157 | 4.70E-06 |
| rs4841899 | C | T | 0.1004 | 0.0178 | 1.78E-08 |
| rs635634 | C | T | 0.1032 | 0.0191 | 6.74E-08 |
| rs72678285 | T | A | -0.1092 | 0.0232 | 2.25E-06 |
| rs78666213 | G | T | 0.2744 | 0.0576 | 2.59E-06 |
| rs80271436 | G | A | 0.237 | 0.0485 | 9.95E-07 |

**Supplementary Table 11** Characteristics of the single nucleotide polymorphisms (SNP) used as instrumental variables for Interleukin-16

| **rsID** | **Effect Allele** | **Other Allele** | **BETA** | **SE** | **P Value** |
| --- | --- | --- | --- | --- | --- |
| rs115981497 | T | A | 0.8306 | 0.1681 | 3.59E-06 |
| rs117217798 | C | T | 0.2036 | 0.0444 | 4.15E-06 |
| rs117916513 | G | A | 0.502 | 0.0986 | 3.79E-07 |
| rs1255143 | C | T | -0.1306 | 0.0242 | 7.10E-08 |
| rs12765671 | G | A | 0.6023 | 0.1318 | 4.84E-06 |
| rs144691581 | G | A | -0.4882 | 0.0967 | 4.20E-07 |
| rs1801020 | G | A | -0.1733 | 0.0272 | 4.53E-10 |
| rs4253283 | C | T | -0.146 | 0.0262 | 1.75E-08 |
| rs4513633 | C | A | 0.2239 | 0.0453 | 7.44E-07 |
| rs4778636 | G | A | 0.7272 | 0.0633 | 1.11E-30 |
| rs9706053 | C | T | -0.4582 | 0.0932 | 7.01E-07 |

**Supplementary Table 12** Characteristics of the single nucleotide polymorphisms (SNP) used as instrumental variables for Regulated on Activation, Normal T Cell Expressed and Secreted (CCL5)

| **rsID** | **Effect Allele** | **Other Allele** | **BETA** | **SE** | **P Value** |
| --- | --- | --- | --- | --- | --- |
| rs112072646 | G | A | -0.4286 | 0.0862 | 6.48E-07 |
| rs147509526 | C | T | 0.358 | 0.0717 | 6.93E-07 |
| rs2251660 | C | A | -0.1829 | 0.0359 | 3.83E-07 |
| rs4940620 | G | A | 0.2494 | 0.054 | 3.54E-06 |
| rs62438851 | G | A | 0.1957 | 0.0414 | 2.33E-06 |
| rs7000423 | C | T | 0.1318 | 0.0253 | 1.82E-07 |
| rs7170339 | G | C | 0.4203 | 0.0912 | 4.41E-06 |
| rs72793342 | G | A | 0.1487 | 0.0308 | 1.48E-06 |
| rs74472919 | C | T | -0.3313 | 0.0605 | 3.97E-08 |
| rs75613039 | C | T | -0.37 | 0.081 | 4.81E-06 |
| rs818452 | C | T | -0.2381 | 0.0505 | 2.36E-06 |

**Supplementary Table 13** Characteristics of the single nucleotide polymorphisms (SNP) used as instrumental variables for Platelet derived growth factor BB (PDGFBB)

| **rsID** | **Effect Allele** | **Other Allele** | **BETA** | **SE** | **P Value** |
| --- | --- | --- | --- | --- | --- |
| rs11247305 | G | C | 0.1692 | 0.0364 | 1.90E-06 |
| rs116445074 | G | T | -0.2931 | 0.0587 | 3.11E-07 |
| rs11766649 | G | A | -0.0908 | 0.0196 | 3.53E-06 |
| rs11916118 | G | A | -0.0889 | 0.0194 | 4.93E-06 |
| rs12289510 | G | A | 0.078 | 0.0158 | 7.69E-07 |
| rs13412535 | G | A | -0.3352 | 0.0214 | 2.46E-55 |
| rs2324229 | C | T | -0.0894 | 0.0161 | 3.48E-08 |
| rs35859699 | G | A | 0.3952 | 0.0842 | 2.07E-06 |
| rs4965869 | C | T | -0.184 | 0.0181 | 5.66E-24 |
| rs55680718 | C | T | 0.1383 | 0.0246 | 1.86E-08 |
| rs72777070 | G | T | 0.1069 | 0.02 | 8.98E-08 |
| rs73162807 | C | A | 0.2391 | 0.0499 | 1.74E-06 |
| rs9936075 | G | A | 0.0782 | 0.0164 | 1.76E-06 |
| rs9941733 | G | A | -0.1161 | 0.0228 | 3.31E-07 |

**Supplementary Table 14** Characteristics of the single nucleotide polymorphisms (SNP) used as instrumental variables for Macrophage inflammatory protein-1β (CCL4)

| **rsID** | **Effect Allele** | **Other Allele** | **BETA** | **SE** | **P Value** |
| --- | --- | --- | --- | --- | --- |
| rs11130043 | G | A | 0.0731 | 0.0157 | 3.22E-06 |
| rs113010081 | C | T | 0.5954 | 0.0236 | 3.85E-140 |
| rs113877493 | C | T | 0.6124 | 0.0218 | 1.62E-173 |
| rs116237296 | G | A | -0.5437 | 0.1115 | 7.23E-07 |
| rs116567227 | G | A | -0.2077 | 0.0336 | 5.33E-10 |
| rs117453826 | G | A | 0.5774 | 0.0593 | 5.07E-22 |
| rs11921171 | C | T | 0.2155 | 0.0471 | 4.44E-06 |
| rs141102180 | G | T | -0.3225 | 0.0393 | 1.08E-16 |
| rs1437220 | C | T | -0.1478 | 0.0315 | 3.53E-06 |
| rs17138331 | G | A | 0.1391 | 0.0295 | 2.26E-06 |
| rs2411161 | C | T | -0.1714 | 0.0367 | 3.14E-06 |
| rs281749 | C | T | -0.0799 | 0.0171 | 3.17E-06 |
| rs3760440 | G | A | -0.1236 | 0.0162 | 2.75E-14 |
| rs4609608 | G | C | 0.0936 | 0.0202 | 3.62E-06 |
| rs72791296 | C | T | -0.2369 | 0.0466 | 3.78E-07 |
| rs72799710 | C | T | 0.1014 | 0.0218 | 3.21E-06 |
| rs74810984 | C | T | -0.2206 | 0.0474 | 1.96E-06 |
| rs76356863 | T | A | 0.3494 | 0.0667 | 1.67E-07 |
| rs76582507 | G | A | -0.3175 | 0.0677 | 3.26E-06 |
| rs76583883 | G | T | 0.2317 | 0.0511 | 4.99E-06 |
| rs76776296 | G | A | -0.2997 | 0.0598 | 5.55E-07 |
| rs79068918 | G | C | -0.2752 | 0.0271 | 3.20E-24 |

**Supplementary Table 15** Characteristics of the single nucleotide polymorphisms (SNP) used as instrumental variables for Macrophage inflammatory protein-1α (CCL3)

| **rsID** | **Effect Allele** | **Other Allele** | **BETA** | **SE** | **P Value** |
| --- | --- | --- | --- | --- | --- |
| rs10835056 | G | T | -0.1194 | 0.0254 | 2.60E-06 |
| rs12690897 | G | A | -0.1248 | 0.0262 | 2.11E-06 |
| rs184154340 | G | A | -0.331 | 0.0693 | 1.86E-06 |
| rs34771762 | G | A | -0.249 | 0.0523 | 2.13E-06 |
| rs57786342 | G | A | -0.1314 | 0.0285 | 4.06E-06 |
| rs60198979 | G | A | 0.2146 | 0.0458 | 2.62E-06 |
| rs6900267 | C | A | 0.2429 | 0.0519 | 2.89E-06 |
| rs7232268 | G | A | -0.2821 | 0.0599 | 2.55E-06 |

**Supplementary Table 16** Characteristics of the single nucleotide polymorphisms (SNP) used as instrumental variables for Monokine induced by interferon-gamma (CXCL9)

| **rsID** | **Effect Allele** | **Other Allele** | **BETA** | **SE** | **P Value** |
| --- | --- | --- | --- | --- | --- |
| rs111607343 | G | A | 0.521 | 0.1119 | 2.83E-06 |
| rs11177248 | G | A | -0.3073 | 0.067 | 4.45E-06 |
| rs112337562 | G | T | 0.37 | 0.0796 | 2.98E-06 |
| rs112861654 | G | A | 0.2765 | 0.0529 | 1.81E-07 |
| rs117831247 | C | T | 0.8334 | 0.1754 | 2.16E-06 |
| rs13143163 | G | C | -0.2706 | 0.0586 | 4.02E-06 |
| rs139010077 | C | T | -0.4322 | 0.095 | 3.55E-06 |
| rs1796086 | C | T | 0.2096 | 0.0403 | 2.23E-07 |
| rs41272086 | G | A | 0.2226 | 0.0415 | 7.43E-08 |
| rs55876513 | G | T | -0.166 | 0.0255 | 8.23E-11 |
| rs5752128 | C | T | 0.1685 | 0.0369 | 4.34E-06 |
| rs62562991 | G | A | -0.6236 | 0.126 | 8.40E-07 |
| rs6679677 | C | A | -0.162 | 0.0329 | 8.86E-07 |
| rs75880256 | G | C | 0.2703 | 0.0586 | 3.89E-06 |
| rs77086208 | C | T | -0.3226 | 0.0698 | 3.83E-06 |
| rs816960 | C | T | 0.1224 | 0.0244 | 5.01E-07 |
| rs902851 | T | A | -0.8205 | 0.1884 | 4.68E-06 |

**Supplementary Table 17** Characteristics of the single nucleotide polymorphisms (SNP) used as instrumental variables for Macrophage colony-stimulating factor (MCSF)

| **rsID** | **Effect Allele** | **Other Allele** | **BETA** | **SE** | **P Value** |
| --- | --- | --- | --- | --- | --- |
| rs116274860 | G | T | -0.819 | 0.1741 | 2.74E-06 |
| rs117867915 | C | T | -0.5272 | 0.1098 | 1.61E-06 |
| rs12962919 | C | T | -0.3052 | 0.0662 | 4.65E-06 |
| rs145778765 | C | T | 0.7993 | 0.1689 | 2.20E-06 |
| rs4269021 | G | C | 0.235 | 0.0507 | 3.66E-06 |
| rs56367447 | C | T | 0.4967 | 0.0883 | 1.72E-08 |
| rs62294910 | G | A | -0.3431 | 0.0691 | 6.82E-07 |
| rs78296352 | G | T | -0.527 | 0.1112 | 1.05E-06 |
| rs9387100 | C | T | 0.1352 | 0.0292 | 4.07E-06 |

**Supplementary Table 18** Characteristics of the single nucleotide polymorphisms (SNP) used as instrumental variables for Monocyte specific chemokine 3 (CCL7)

| **rsID** | **Effect Allele** | **Other Allele** | **BETA** | **SE** | **P Value** |
| --- | --- | --- | --- | --- | --- |
| rs10892381 | C | T | -0.2412 | 0.0476 | 3.56E-07 |
| rs28394764 | T | A | -0.6012 | 0.1282 | 3.01E-06 |
| rs62492260 | G | T | 0.2788 | 0.058 | 1.54E-06 |
| rs73669117 | G | A | 0.6238 | 0.131 | 2.56E-06 |

**Supplementary Table 19** Characteristics of the single nucleotide polymorphisms (SNP) used as instrumental variables for Monocyte chemotactic protein-1 (CCL2)

| **rsID** | **Effect Allele** | **Other Allele** | **BETA** | **SE** | **P Value** |
| --- | --- | --- | --- | --- | --- |
| rs10744620 | C | T | -0.0788 | 0.0161 | 9.91E-07 |
| rs10888395 | C | T | 0.0814 | 0.0163 | 5.98E-07 |
| rs111995966 | G | T | -0.1452 | 0.031 | 2.53E-06 |
| rs12073356 | G | A | 0.1426 | 0.0311 | 4.17E-06 |
| rs12075 | G | A | -0.2185 | 0.0155 | 1.44E-44 |
| rs146522229 | C | T | 0.5976 | 0.1177 | 3.56E-07 |
| rs2036297 | G | A | -0.119 | 0.016 | 1.09E-13 |
| rs2288370 | C | T | 0.1031 | 0.0163 | 2.25E-10 |
| rs2712431 | C | A | 0.0787 | 0.0172 | 4.76E-06 |
| rs56212190 | C | T | -0.181 | 0.0373 | 9.85E-07 |
| rs7197349 | G | A | -0.0968 | 0.0206 | 2.62E-06 |
| rs7517040 | G | A | 0.0987 | 0.0191 | 2.44E-07 |
| rs7632755 | G | A | -0.2938 | 0.0316 | 1.18E-20 |
| rs9317045 | C | A | -0.1134 | 0.0236 | 1.52E-06 |

**Supplementary Table 20** Characteristics of the single nucleotide polymorphisms (SNP) used as instrumental variables for Interleukin-12p70

| **rsID** | **Effect Allele** | **Other Allele** | **BETA** | **SE** | **P Value** |
| --- | --- | --- | --- | --- | --- |
| rs10761731 | T | A | 0.1001 | 0.0162 | 6.23E-10 |
| rs13209117 | G | A | -0.1002 | 0.0186 | 5.57E-08 |
| rs17229494 | G | A | 0.1172 | 0.0257 | 4.93E-06 |
| rs2375980 | G | C | -0.0937 | 0.0159 | 4.55E-09 |
| rs282258 | C | T | -0.073 | 0.0156 | 3.21E-06 |
| rs41282644 | G | A | -0.1473 | 0.0304 | 1.05E-06 |
| rs4349809 | G | T | -0.3777 | 0.0159 | 2.56E-124 |
| rs6993770 | T | A | -0.0918 | 0.0189 | 1.12E-06 |
| rs71361173 | G | T | -0.111 | 0.0239 | 3.06E-06 |
| rs782107 | G | A | -0.075 | 0.0156 | 1.60E-06 |
| rs79121401 | C | T | -0.5548 | 0.1206 | 4.24E-06 |

**Supplementary Table 21** Characteristics of the single nucleotide polymorphisms (SNP) used as instrumental variables for Interferon gamma-induced protein 10 (CXCL10)

| **rsID** | **Effect Allele** | **Other Allele** | **BETA** | **SE** | **P Value** |
| --- | --- | --- | --- | --- | --- |
| rs10809307 | C | T | -0.1305 | 0.0282 | 3.64E-06 |
| rs113831257 | G | A | -0.3592 | 0.0644 | 2.53E-08 |
| rs11626201 | C | A | -0.1162 | 0.0245 | 1.93E-06 |
| rs143799975 | G | A | 0.7984 | 0.1637 | 1.00E-06 |
| rs188759467 | T | A | -0.4504 | 0.0964 | 3.22E-06 |
| rs34383175 | C | T | 0.3153 | 0.0657 | 1.51E-06 |
| rs397816 | C | T | -0.1237 | 0.0249 | 7.90E-07 |
| rs75970138 | G | A | 0.485 | 0.104 | 1.53E-06 |
| rs7645625 | G | T | 0.1086 | 0.0237 | 4.41E-06 |
| rs79848609 | C | A | -0.2603 | 0.0537 | 8.75E-07 |
| rs8112909 | G | A | 0.1426 | 0.0299 | 1.94E-06 |
| rs9450351 | C | T | 0.2768 | 0.0489 | 1.48E-08 |

**Supplementary Table 22** Characteristics of the single nucleotide polymorphisms (SNP) used as instrumental variables for Interleukin-18

| **rsID** | **Effect Allele** | **Other Allele** | **BETA** | **SE** | **P Value** |
| --- | --- | --- | --- | --- | --- |
| rs10414578 | C | T | 0.1771 | 0.035 | 4.16E-07 |
| rs115267715 | C | T | -0.4508 | 0.08 | 1.72E-08 |
| rs116383510 | C | A | 0.5426 | 0.1056 | 3.00E-07 |
| rs11700536 | C | T | -0.1156 | 0.025 | 4.21E-06 |
| rs117266781 | C | T | -0.6841 | 0.1468 | 3.15E-06 |
| rs143370787 | G | C | 0.3116 | 0.066 | 2.35E-06 |
| rs144841621 | C | T | -0.518 | 0.1141 | 3.81E-06 |
| rs1656939 | T | A | -0.1132 | 0.0236 | 1.56E-06 |
| rs17229943 | C | A | 0.312 | 0.0463 | 1.62E-11 |
| rs1852105 | C | T | -0.3036 | 0.0661 | 4.32E-06 |
| rs1979967 | C | T | -0.1402 | 0.0286 | 9.45E-07 |
| rs2729385 | G | A | -0.1231 | 0.0262 | 3.79E-06 |
| rs385076 | C | T | 0.2432 | 0.0248 | 1.66E-22 |
| rs4482818 | G | A | -0.1286 | 0.0244 | 1.45E-07 |
| rs658805 | G | A | -0.1226 | 0.0244 | 4.94E-07 |
| rs71478720 | C | T | 0.2669 | 0.0276 | 3.71E-22 |
| rs78623212 | C | T | -0.8705 | 0.1778 | 6.71E-07 |
| rs78716465 | G | A | -0.3265 | 0.0682 | 1.63E-06 |

**Supplementary Table 23** Characteristics of the single nucleotide polymorphisms (SNP) used as instrumental variables for Interleukin-17

| **rsID** | **Effect Allele** | **Other Allele** | **BETA** | **SE** | **P Value** |
| --- | --- | --- | --- | --- | --- |
| rs11640734 | G | C | 0.1187 | 0.0241 | 5.35E-07 |
| rs117029961 | G | A | -0.4585 | 0.1015 | 4.94E-06 |
| rs117556572 | C | T | 0.5102 | 0.1099 | 3.28E-06 |
| rs148562661 | G | C | -0.2128 | 0.0435 | 1.00E-06 |
| rs1530455 | C | T | -0.108 | 0.0173 | 4.87E-10 |
| rs17106604 | C | T | -0.1129 | 0.0225 | 6.37E-07 |
| rs17282552 | C | T | 0.2001 | 0.0405 | 8.21E-07 |
| rs184080173 | C | T | -0.2384 | 0.0471 | 4.19E-07 |
| rs187475560 | C | T | 0.2434 | 0.052 | 3.29E-06 |
| rs62191444 | G | T | 0.1136 | 0.0247 | 4.22E-06 |
| rs78296352 | G | T | -0.3027 | 0.0646 | 4.27E-06 |
| rs78612928 | C | T | -0.1037 | 0.0222 | 2.62E-06 |

**Supplementary Table 24** Characteristics of the single nucleotide polymorphisms (SNP) used as instrumental variables for Interleukin-13

| **rsID** | **Effect Allele** | **Other Allele** | **BETA** | **SE** | **P Value** |
| --- | --- | --- | --- | --- | --- |
| rs117795020 | G | A | 0.3522 | 0.0716 | 9.86E-07 |
| rs12623722 | G | A | 0.1185 | 0.0258 | 4.19E-06 |
| rs139083458 | C | T | -0.9902 | 0.2107 | 2.81E-06 |
| rs142167313 | C | T | 0.313 | 0.0617 | 3.98E-07 |
| rs2370048 | G | A | 0.1235 | 0.0266 | 3.35E-06 |
| rs27949 | C | T | 0.1168 | 0.0252 | 3.43E-06 |
| rs6799107 | C | T | 0.1459 | 0.0301 | 1.25E-06 |
| rs7073807 | C | T | -0.1682 | 0.0356 | 2.37E-06 |
| rs75383097 | G | C | 0.536 | 0.1165 | 4.29E-06 |
| rs75995699 | G | A | -0.3319 | 0.0698 | 2.64E-06 |
| rs76339001 | T | A | 0.4313 | 0.0886 | 1.15E-06 |
| rs9472168 | G | A | -0.4244 | 0.0248 | 1.08E-65 |

**Supplementary Table 25** Characteristics of the single nucleotide polymorphisms (SNP) used as instrumental variables for Interleukin-10

| **rsID** | **Effect Allele** | **Other Allele** | **BETA** | **SE** | **P Value** |
| --- | --- | --- | --- | --- | --- |
| rs10457128 | G | A | 0.0865 | 0.0172 | 5.24E-07 |
| rs10493718 | C | A | 0.11 | 0.0222 | 7.16E-07 |
| rs111913416 | T | A | 0.085 | 0.0172 | 7.23E-07 |
| rs11206302 | C | T | 0.1189 | 0.0251 | 2.20E-06 |
| rs2086656 | C | T | 0.0789 | 0.0171 | 3.78E-06 |
| rs2375980 | G | C | -0.0824 | 0.0165 | 7.07E-07 |
| rs282258 | C | T | -0.0992 | 0.0162 | 1.00E-09 |
| rs3025021 | C | T | -0.0947 | 0.0195 | 1.46E-06 |
| rs41282660 | G | A | 0.1194 | 0.0255 | 3.72E-06 |
| rs4349809 | G | T | -0.2853 | 0.0165 | 5.77E-67 |
| rs465757 | G | A | -0.084 | 0.0174 | 1.17E-06 |
| rs6085948 | G | A | -0.098 | 0.0202 | 1.25E-06 |
| rs7088799 | G | T | 0.0852 | 0.0167 | 3.23E-07 |

**Supplementary Table 26** Characteristics of the single nucleotide polymorphisms (SNP) used as instrumental variables for Interleukin-8 (CXCL8)

| **rsID** | **Effect Allele** | **Other Allele** | **BETA** | **SE** | **P Value** |
| --- | --- | --- | --- | --- | --- |
| rs11634944 | C | T | 0.1214 | 0.0252 | 1.29E-06 |
| rs12075 | G | A | -0.12 | 0.0236 | 3.88E-07 |
| rs141926526 | C | A | 0.6149 | 0.1308 | 2.57E-06 |
| rs2673604 | C | A | 0.1266 | 0.0255 | 7.02E-07 |

**Supplementary Table 27** Characteristics of the single nucleotide polymorphisms (SNP) used as instrumental variables for Interleukin-6

| **rsID** | **Effect Allele** | **Other Allele** | **BETA** | **SE** | **P Value** |
| --- | --- | --- | --- | --- | --- |
| rs10752777 | T | A | -0.1098 | 0.0236 | 3.25E-06 |
| rs1333040 | C | T | -0.0738 | 0.0158 | 3.17E-06 |
| rs13412535 | G | A | 0.1164 | 0.0215 | 7.34E-08 |
| rs1884910 | G | C | -0.079 | 0.0169 | 3.26E-06 |
| rs73273528 | C | T | -0.2672 | 0.0553 | 9.58E-07 |
| rs75101555 | G | C | 0.3667 | 0.0781 | 2.60E-06 |
| rs76856708 | C | T | -0.3289 | 0.07 | 2.61E-06 |

**Supplementary Table 28** Characteristics of the single nucleotide polymorphisms (SNP) used as instrumental variables for Interleukin-1 receptor antagonist

| **rsID** | **Effect Allele** | **Other Allele** | **BETA** | **SE** | **P Value** |
| --- | --- | --- | --- | --- | --- |
| rs1054402 | C | T | -0.1311 | 0.027 | 1.13E-06 |
| rs11627423 | C | A | -0.1171 | 0.0247 | 2.12E-06 |
| rs11869294 | G | C | 0.2274 | 0.0474 | 1.50E-06 |
| rs12121840 | C | T | -0.2692 | 0.0571 | 2.43E-06 |
| rs2809154 | C | T | 0.1786 | 0.0388 | 3.74E-06 |
| rs61335305 | C | A | -0.4453 | 0.0908 | 1.00E-06 |
| rs9623661 | C | T | 0.1966 | 0.0426 | 3.86E-06 |

**Supplementary Table 29** Characteristics of the single nucleotide polymorphisms (SNP) used as instrumental variables for Interleukin-1-beta

| **rsID** | **Effect Allele** | **Other Allele** | **BETA** | **SE** | **P Value** |
| --- | --- | --- | --- | --- | --- |
| rs143319329 | C | T | -0.2801 | 0.0715 | 2.00E-06 |
| rs1942793 | G | T | -0.0717 | 0.0187 | 4.98E-06 |
| rs61335305 | C | A | -0.2966 | 0.0724 | 1.90E-06 |
| rs62015704 | G | A | -0.1082 | 0.0283 | 2.09E-06 |
| rs9898641 | C | T | 0.2032 | 0.0454 | 3.59E-06 |

**Supplementary Table 30** Characteristics of the single nucleotide polymorphisms (SNP) used as instrumental variables for Hepatocyte growth factor

| **rsID** | **Effect Allele** | **Other Allele** | **BETA** | **SE** | **P Value** |
| --- | --- | --- | --- | --- | --- |
| rs11060254 | G | A | 0.08 | 0.0167 | 1.58E-06 |
| rs150322232 | G | A | -0.2104 | 0.0463 | 4.89E-06 |
| rs1698249 | C | A | 0.1698 | 0.0372 | 4.09E-06 |
| rs180840563 | T | A | 0.2002 | 0.0416 | 1.53E-06 |
| rs2003620 | C | T | -0.2279 | 0.0489 | 2.83E-06 |
| rs3748034 | G | T | -0.1495 | 0.0234 | 1.81E-10 |
| rs5745687 | C | T | 0.3072 | 0.0406 | 2.75E-14 |
| rs6077285 | G | C | 0.1393 | 0.0274 | 3.20E-07 |
| rs62481625 | C | T | -0.1091 | 0.0225 | 1.18E-06 |

**Supplementary Table 31** Characteristics of the single nucleotide polymorphisms (SNP) used as instrumental variables for Interleukin-9

| **rsID** | **Effect Allele** | **Other Allele** | **BETA** | **SE** | **P Value** |
| --- | --- | --- | --- | --- | --- |
| rs117807175 | G | C | 0.5143 | 0.1113 | 4.29E-06 |
| rs3736858 | G | C | 0.1374 | 0.0293 | 2.76E-06 |
| rs41294750 | C | T | -0.3514 | 0.0748 | 2.37E-06 |
| rs4880409 | C | T | 0.3355 | 0.0723 | 3.50E-06 |
| rs61867538 | C | T | -0.3566 | 0.0774 | 3.93E-06 |
| rs7232268 | G | A | -0.2759 | 0.0587 | 2.53E-06 |
| rs7242404 | G | A | 0.1228 | 0.0264 | 3.27E-06 |
| rs76963786 | C | T | 0.2865 | 0.0557 | 4.50E-07 |

**Supplementary Table 32** Characteristics of the single nucleotide polymorphisms (SNP) used as instrumental variables for Interleukin-7

| **rsID** | **Effect Allele** | **Other Allele** | **BETA** | **SE** | **P Value** |
| --- | --- | --- | --- | --- | --- |
| rs117509142 | C | T | 0.327 | 0.0688 | 1.99E-06 |
| rs141425475 | C | T | 0.4781 | 0.1016 | 2.53E-06 |
| rs144701438 | G | A | 0.4819 | 0.0989 | 9.75E-07 |
| rs17091524 | C | T | -0.4924 | 0.1013 | 1.91E-06 |
| rs218247 | G | C | 0.1352 | 0.0286 | 2.31E-06 |
| rs28793375 | C | T | -0.1638 | 0.0361 | 4.46E-06 |
| rs4320361 | G | T | 0.3245 | 0.0249 | 6.87E-39 |
| rs75904417 | C | A | 0.1698 | 0.0349 | 1.16E-06 |
| rs77981494 | C | T | 0.5178 | 0.1064 | 1.07E-06 |
| rs78346957 | G | A | -0.4588 | 0.1007 | 4.51E-06 |
| rs8175379 | T | A | -0.1307 | 0.0285 | 4.38E-06 |

**Supplementary Table 33** Characteristics of the single nucleotide polymorphisms (SNP) used as instrumental variables for Interleukin-5

| **rsID** | **Effect Allele** | **Other Allele** | **BETA** | **SE** | **P Value** |
| --- | --- | --- | --- | --- | --- |
| rs11680908 | G | A | -0.2634 | 0.0554 | 2.03E-06 |
| rs6737109 | C | T | -0.116 | 0.0247 | 2.40E-06 |
| rs72831687 | G | A | 0.5239 | 0.1109 | 1.69E-06 |
| rs73040130 | C | T | -0.2638 | 0.0529 | 6.00E-07 |
| rs7578892 | G | C | 0.1173 | 0.0249 | 2.44E-06 |
| rs7767396 | G | A | -0.1515 | 0.0246 | 7.69E-10 |

**Supplementary Table 34** Characteristics of the single nucleotide polymorphisms (SNP) used as instrumental variables for Interleukin-4

| **rsID** | **Effect Allele** | **Other Allele** | **BETA** | **SE** | **P Value** |
| --- | --- | --- | --- | --- | --- |
| rs10512267 | C | T | 0.0824 | 0.0161 | 2.94E-07 |
| rs116705532 | G | T | 0.4678 | 0.0978 | 1.76E-06 |
| rs117146485 | C | T | 0.2924 | 0.0629 | 2.71E-06 |
| rs17713451 | G | A | -0.1274 | 0.0253 | 4.97E-07 |
| rs4444693 | T | A | -0.0881 | 0.0184 | 1.70E-06 |
| rs73023729 | G | A | 0.1796 | 0.0366 | 9.03E-07 |
| rs7613691 | G | A | -0.1775 | 0.0384 | 4.05E-06 |
| rs79597994 | C | T | 0.5831 | 0.127 | 4.32E-06 |
| rs9508291 | C | T | 0.1676 | 0.0359 | 3.03E-06 |
| rs9941733 | G | A | -0.114 | 0.0229 | 6.88E-07 |

**Supplementary Table 35** Characteristics of the single nucleotide polymorphisms (SNP) used as instrumental variables for Interleukin-2 receptor, alpha subunit

| **rsID** | **Effect Allele** | **Other Allele** | **BETA** | **SE** | **P Value** |
| --- | --- | --- | --- | --- | --- |
| rs11241559 | G | T | 0.1264 | 0.0266 | 2.00E-06 |
| rs115360066 | G | A | -0.1867 | 0.0379 | 8.06E-07 |
| rs117244812 | G | A | 0.7064 | 0.1488 | 2.10E-06 |
| rs12722497 | C | A | -0.6279 | 0.0485 | 1.57E-38 |
| rs185231391 | C | T | -0.8503 | 0.1809 | 1.47E-06 |
| rs4733117 | C | A | -0.1369 | 0.0292 | 2.63E-06 |
| rs61705228 | C | T | -0.3303 | 0.0716 | 3.99E-06 |
| rs759244 | T | A | 0.1126 | 0.0239 | 2.43E-06 |
| rs929551 | G | C | -0.1686 | 0.0352 | 1.61E-06 |

**Supplementary Table 36** Characteristics of the single nucleotide polymorphisms (SNP) used as instrumental variables for Interleukin-2

| **rsID** | **Effect Allele** | **Other Allele** | **BETA** | **SE** | **P Value** |
| --- | --- | --- | --- | --- | --- |
| rs12051139 | C | T | 0.1131 | 0.0247 | 4.76E-06 |
| rs13412535 | G | A | -0.1764 | 0.0332 | 1.18E-07 |
| rs170117 | C | T | 0.1617 | 0.0349 | 3.87E-06 |
| rs2807544 | G | A | -0.1175 | 0.0253 | 3.41E-06 |
| rs4634519 | G | A | 0.1261 | 0.0269 | 2.77E-06 |
| rs61335305 | C | A | -0.4514 | 0.0918 | 7.32E-07 |
| rs62124990 | G | T | 0.6961 | 0.1495 | 3.22E-06 |
| rs7615304 | G | A | 0.1172 | 0.0242 | 1.21E-06 |
| rs7806875 | T | A | -0.1192 | 0.0248 | 1.47E-06 |
| rs80336398 | C | T | -0.4001 | 0.0858 | 2.82E-06 |

**Supplementary Table 37** Characteristics of the single nucleotide polymorphisms (SNP) used as instrumental variables for Interferon-gamma

| **rsID** | **Effect Allele** | **Other Allele** | **BETA** | **SE** | **P Value** |
| --- | --- | --- | --- | --- | --- |
| rs10487554 | G | A | 0.0895 | 0.0183 | 1.09E-06 |
| rs10761731 | T | A | 0.0826 | 0.0167 | 8.09E-07 |
| rs112783231 | G | A | 0.2408 | 0.0511 | 1.96E-06 |
| rs115729819 | G | A | -0.2484 | 0.0515 | 1.38E-06 |
| rs11843756 | G | T | -0.184 | 0.0393 | 3.09E-06 |
| rs12420286 | C | T | -0.2376 | 0.0501 | 2.08E-06 |
| rs1867282 | C | T | -0.0774 | 0.0166 | 3.15E-06 |
| rs2073438 | G | A | -0.0898 | 0.0188 | 1.68E-06 |
| rs2188420 | G | C | -0.0987 | 0.0202 | 1.03E-06 |
| rs73479333 | G | C | 0.1159 | 0.024 | 1.34E-06 |
| rs74148555 | C | T | 0.3732 | 0.0774 | 2.64E-06 |
| rs78296352 | G | T | -0.343 | 0.0652 | 1.38E-07 |

**Supplementary Table 38** Characteristics of the single nucleotide polymorphisms (SNP) used as instrumental variables for Growth regulated oncogene-α (CXCL1)

| **rsID** | **Effect Allele** | **Other Allele** | **BETA** | **SE** | **P Value** |
| --- | --- | --- | --- | --- | --- |
| rs1113500 | G | T | -0.1174 | 0.0244 | 1.57E-06 |
| rs118158560 | G | A | -0.2703 | 0.0594 | 3.42E-06 |
| rs12075 | G | A | -0.3751 | 0.0237 | 1.24E-55 |
| rs140734053 | G | A | -0.7257 | 0.1561 | 3.58E-06 |
| rs185768063 | G | A | -0.3998 | 0.076 | 1.46E-07 |
| rs188345231 | C | T | -0.623 | 0.1323 | 4.34E-06 |
| rs2422841 | G | A | 0.1657 | 0.0361 | 4.66E-06 |
| rs508977 | G | T | 0.3802 | 0.028 | 7.56E-42 |
| rs62024303 | G | A | 0.3053 | 0.0666 | 4.41E-06 |
| rs78653452 | G | T | 0.7362 | 0.1558 | 1.21E-06 |

**Supplementary Table 39** Characteristics of the single nucleotide polymorphisms (SNP) used as instrumental variables for Granulocyte colony-stimulating factor

| **rsID** | **Effect Allele** | **Other Allele** | **BETA** | **SE** | **P Value** |
| --- | --- | --- | --- | --- | --- |
| rs115256310 | G | A | 0.6821 | 0.136 | 6.73E-07 |
| rs11903143 | G | A | -0.087 | 0.0176 | 6.35E-07 |
| rs139692213 | T | A | 0.6028 | 0.1336 | 4.80E-06 |
| rs145756094 | G | C | 0.7408 | 0.1479 | 5.53E-07 |
| rs147128865 | C | T | -0.27 | 0.0587 | 4.92E-06 |
| rs1817411 | C | T | -0.089 | 0.0191 | 3.10E-06 |
| rs2324653 | G | C | 0.0808 | 0.0161 | 5.03E-07 |
| rs2671444 | G | A | 0.0784 | 0.0166 | 2.48E-06 |
| rs74148555 | C | T | 0.3715 | 0.0755 | 1.55E-06 |
| rs76287671 | C | T | -0.0938 | 0.0189 | 6.92E-07 |
| rs77318030 | C | T | 0.2045 | 0.0428 | 2.21E-06 |

**Supplementary Table 40** Characteristics of the single nucleotide polymorphisms (SNP) used as instrumental variables for Basic fibroblast growth factor

| **rsID** | **Effect Allele** | **Other Allele** | **BETA** | **SE** | **P Value** |
| --- | --- | --- | --- | --- | --- |
| rs116920326 | G | C | 0.2435 | 0.0534 | 4.95E-06 |
| rs13412535 | G | A | 0.1112 | 0.0225 | 7.35E-07 |
| rs145577605 | G | A | -0.2081 | 0.0428 | 9.64E-07 |
| rs16873997 | G | C | 0.4922 | 0.1048 | 2.99E-06 |
| rs61990749 | G | C | -0.1116 | 0.0229 | 1.24E-06 |
| rs747334 | G | A | -0.0751 | 0.0164 | 4.53E-06 |
| rs75168112 | C | T | 0.1001 | 0.0214 | 3.00E-06 |
| rs9907295 | C | T | 0.1319 | 0.0269 | 7.95E-07 |

**Supplementary Table 41** Characteristics of the single nucleotide polymorphisms (SNP) used as instrumental variables for Eotaxin (CCL11)

| **rsID** | **Effect Allele** | **Other Allele** | **BETA** | **SE** | **P Value** |
| --- | --- | --- | --- | --- | --- |
| rs112347425 | C | T | -0.158 | 0.0277 | 8.65E-09 |
| rs12075 | G | A | -0.1671 | 0.0156 | 1.33E-26 |
| rs1476670 | C | A | 0.1007 | 0.0217 | 3.51E-06 |
| rs187131 | G | C | -0.1291 | 0.0254 | 3.57E-07 |
| rs2024050 | G | A | -0.1728 | 0.0303 | 1.10E-08 |
| rs2210755 | C | T | 0.1104 | 0.0242 | 4.85E-06 |
| rs2211994 | C | T | -0.0885 | 0.0177 | 6.08E-07 |
| rs2228467 | C | T | 0.4163 | 0.0292 | 2.27E-46 |
| rs2419841 | C | T | 0.1277 | 0.0279 | 4.98E-06 |
| rs5746492 | G | A | -0.0954 | 0.0207 | 3.96E-06 |
| rs5754733 | C | A | 0.1042 | 0.0214 | 1.06E-06 |
| rs59808887 | C | T | 0.1673 | 0.0358 | 2.91E-06 |
| rs75426604 | C | A | 0.1366 | 0.0291 | 2.53E-06 |
| rs79722574 | C | T | 0.1113 | 0.0228 | 1.06E-06 |
| rs80341932 | G | A | -0.1016 | 0.0205 | 6.69E-07 |
| rs9317045 | C | A | -0.1182 | 0.0237 | 5.82E-07 |

**Supplementary Table 42** Characteristics of the single nucleotide polymorphisms (SNP) used as instrumental variables for Meningiomas

| **rsID** | **Effect Allele** | **Other Allele** | **BETA** | **SE** | **P Value** |
| --- | --- | --- | --- | --- | --- |
| rs10833988 | A | G | 0.415964 | 0.0896314 | 3.47E-06 |
| rs146864255 | T | C | 2.67588 | 0.581002 | 4.11E-06 |
| rs17033809 | A | C | 0.534657 | 0.114792 | 3.20E-06 |
| rs192521781 | T | C | 2.60899 | 0.544207 | 1.63E-06 |
| rs289744 | G | T | 0.420115 | 0.0907327 | 3.65E-06 |
| rs56895157 | C | T | 1.13682 | 0.248273 | 4.67E-06 |
| rs73944983 | A | G | 1.60717 | 0.347488 | 3.74E-06 |
| rs78907823 | G | A | 0.746534 | 0.153739 | 1.20E-06 |
| rs9365501 | G | T | -0.440217 | 0.0915552 | 1.52E-06 |
| rs9961255 | C | T | 0.423611 | 0.0894279 | 2.17E-06 |
